# To Remove Rust

**Published:** 1892-11

**Authors:** Mart L. Brodie

**Affiliations:** Ray, Ky.


### To Remove Rust.

If Dr. Fridge will take a saturated solution of chloride of tin in distilled water, and fill (sufficiently full to admit the instrument) a large test tube; let the instrument remain over night, and next morning, rub dry with a chamois, after rinsing in running water, the instrument will be of a neat silver whiteness.

Ray, Ky. Mart L. Brodie, M.D.

### Boils.

Boils may be aborted, says an exchange, by energetically painting them several times a day with strong tincture of iodine. The application should be made strong and frequent enough to make the painted portion appear black.

